# Diseases Tracked by Using Google Trends, Spain

**DOI:** 10.3201/eid1601.091308

**Published:** 2010-01

**Authors:** Antonio Valdivia, Susana Monge-Corella

**Affiliations:** Hospital Universitario de La Princesa, Madrid, Spain (A. Valdivia); Instituto de Salud Carlos III, Madrid (S. Monge-Corella).; 1 These authors contributed equally to this article.

**Keywords:** Internet, epidemiologic surveillance, chickenpox, influenza, viruses, human, letter

**To the Editor:** We read the article by Pelat et al. (*1*) with great interest and decided to explore whether this tool could be applicable for non-English and non-French speaking countries and, more specifically, for Spain. We compared the Google queries related to influenza-like illness (ILI) and chickenpox described by Pelat et al. (*1*), and constructed additional queries with symptoms and conditions frequently associated with ILI.

The weekly queries from January 2004 through February 2009 were downloaded from Google Insights for Search (*2*). We studied the correlation (Spearman ρ) of these queries with the data from the national reporting of notifiable diseases, available from the Spanish National Epidemiology Center website (*3*), assuming a maximum difference of 4 weeks.

The queries for *gripe* (Spanish for influenza) showed a maximum correlation (ρ = 0.70) 2 weeks before the declared ILI (DILI). When excluding the terms for *aviar* (avian) and *vacuna* (vaccine), the correlation peak (ρ = 0.81) was likewise observable 2 weeks before the DILI. The maximum correlation observed for symptom queries was for *tos* (Spanish for cough) 2 weeks before the DILI (ρ = 0.74); for conditions associated with influenza the correlation was for *neumonía* (Spanish for pneumonia, accented or unaccented) 2 weeks after the DILI (ρ = 0.84). The queries for *varicela* (Spanish for chickenpox) showed a maximum correlation (ρ = 0.96) 1 week after the declared illness, as observed by Pelat et al (*1*).

In conclusion, our study points out the utility of Internet queries for the surveillance of ILI and chickenpox in Spain. In the case of ILI, this information can be used as an early warning tool used complementarily to standard surveillance systems. More detailed studies are necessary regarding the usefulness and limitations of this tool in Spain, as well as in other contexts.
